# Insights into the mechanisms of angiogenesis in hepatoblastoma

**DOI:** 10.3389/fcell.2025.1535339

**Published:** 2025-05-14

**Authors:** Meng Kong, Yunpeng Zhai, Hongzhen Liu, Shisong Zhang, Shuai Chen, Wenfei Li, Xiang Ma, Yi Ji

**Affiliations:** ^1^ Department of Pediatric Surgery, Children’s Hospital Affiliated to Shandong University, Jinan, China; ^2^ Department of Pediatric Surgery, Jinan Children’s Hospital, Jinan, China; ^3^ School of Basic Medical Sciences, Cheeloo College of Medicine, Shandong University, Jinan, China; ^4^ Department of Respiratory Disease, Children’s Hospital Affiliated to Shandong University, Jinan, China; ^5^ Jinan Key Laboratory of Pediatric Respiratory Diseases, Jinan Children’s Hospital, Jinan, China; ^6^ Division of Oncology, Department of Pediatric Surgery, West China Hospital, Sichuan University, Chengdu, China

**Keywords:** hepatoblastoma, angiogenesis, signalling pathways, molecular mechanisms, treatment strategies

## Abstract

Hepatoblastoma (HB), the most common pediatric liver malignancy, is characterized by aggressive growth and metastasis driven by complex angiogenic mechanisms. This review elucidates the pivotal role of angiogenesis in HB progression, emphasizing metabolic reprogramming, tumor microenvironment (TME) dynamics, and oncogenic signalling pathways. The Warburg effect in HB cells fosters a hypoxic microenvironment, stabilizing hypoxia-inducible factor-1α (HIF-1α) and upregulating vascular endothelial growth factor (VEGF), which synergistically enhances angiogenesis. Key pathways such as the Wnt/β-catenin, VEGF, PI3K/AKT, and JAK2/STAT3 pathways are central to endothelial cell proliferation, migration, and vascular maturation, whereas interactions with tumor-associated macrophages (TAMs) and pericytes further remodel the TME to support neovascularization. Long noncoding RNAs and glycolytic enzymes have emerged as critical regulators of angiogenesis, linking metabolic activity with vascular expansion. Anti-angiogenic therapies, including VEGF inhibitors and metabolic pathway-targeting agents, show preclinical promise but face challenges such as resistance and off-target effects. Future directions advocate for dual-target strategies, spatial multiomics technologies to map metabolic–angiogenic crosstalk, and personalized approaches leveraging biomarkers for risk stratification. This synthesis underscores the need for interdisciplinary collaboration to translate mechanistic insights into durable therapies, ultimately improving outcomes for HB patients.

## 1 Introduction

Hepatoblastoma (HB) is the most common primary malignant liver tumor in early childhood, accounting for 80% of pediatric liver malignancies and 1% of all childhood malignancies (Wang H. S. et al., 2024). Most HBs are sporadic and commonly occur in children under 5 years of age, predominantly boys (Spector and Birch, 2012). According to monitoring and epidemiological studies, the incidence of HB in the United States is 1.5–1.9 cases per million, showing an increasing trend (Sivaprakasam et al., 2011). This is similar to the incidence in China, which is approximately 1.1 cases per million, and in Nordic countries, which is 1.7 cases per million (Chen et al., 2021). Although HB is relatively rare, it remains a significant disease that poses a serious threat to children’s lives because of its high mortality rate (Feng et al., 2019). In addition, Racial differences are related to incidence rates. Reports indicate that the incidence is relatively low among black individuals (Friedrich et al., 2017). A population-based analysis in the United States revealed that the higher the educational level of the mother was, the lower the incidence of HB was (Kehm et al., 2018). The occurrence of HB is also associated with certain genetic and environmental factors, particularly in children with a family history, where the incidence is relatively high (Liu et al., 2020). Additionally, HB is significantly associated with certain congenital diseases, such as Wilms tumor and phenylketonuria, suggesting a possible common pathogenic mechanism (Castle et al., 2022). Although HB is relatively rare, it remains a significant disease that poses a serious threat to children’s lives because of its high mortality rate (Feng et al., 2019; Honda et al., 2023). Current studies indicate that angiogenesis plays a crucial role in tumor development (Liu et al., 2023). Angiogenesis not only provides the necessary oxygen and nutrients to tumor cells but also promotes tumor metastasis and aggressive growth, making it particularly important to explore the relationship between HB and angiogenesis.

In recent years, an increasing number of studies have focused on the angiogenic characteristics of HB. Research has shown that various angiogenesis-related factors can be observed in HB cells, which enhance the tumor blood supply by promoting the proliferation and migration of endothelial cells, thereby supporting rapid tumor growth and metastasis (Muench et al., 2023). Additionally, some studies have noted that specific gene mutations in HBs may be closely related to angiogenesis, providing new insights into the biological characteristics of HBs (Ma et al., 2024). In this contest, angiogenesis inhibition is considered an effective strategy for antitumor therapy. By targeting angiogenesis, researchers hope to improve the prognosis of HB patients (Martinelli and Ciardiello, 2023). Currently, individualized chemotherapy regimens have been shown to be effective in HB patients, especially when combined with antiangiogenic drugs (Hu et al., 2024). Therefore, exploring the relationship between HB and angiogenesis not only enriches our understanding of tumor biology but also provides new ideas and directions for future treatment strategies.

## 2 Basic mechanism of angiogenesis

### 2.1 Definition and process of angiogenesis

Angiogenesis refers to the process of developing new blood vessels from existing capillaries, ultimately forming a complete, organized, and mature vascular network. This process plays a crucial role in both physiological and pathological states (Larionova et al., 2021). In normal physiological processes, angiogenesis is essential for muscle growth, embryonic development, tissue repair, wound healing, and regeneration of the organ endothelium. During wound healing and fetal development, angiogenesis provides the necessary oxygen and nutrients to support cell proliferation and functional recovery (Duan et al., 2024). In pathological conditions, such as tumors, diabetes, and cardiovascular diseases, abnormal angiogenesis can lead to disease progression and deterioration (Thorin et al., 2023). The mechanisms of angiogenesis involve multiple steps, including the activation, proliferation, migration, and lumen formation of endothelial cells (Augustin and Koh, 2024). Under the stimulation of growth factors, endothelial cells undergo a transition from a quiescent state to a proliferative and migratory state, which is regulated by various cytokines, signalling pathways, and extracellular matrix components, such as vascular endothelial growth factor A (VEGF-A) and fibroblast growth factor 2 (FGF-2) (Zhao et al., 2022; Claffey et al., 2001). Changes in the microenvironment and remodelling of matrix components also play significant roles in angiogenesis (Ionescu et al., 2022).

**FIGURE 1:**
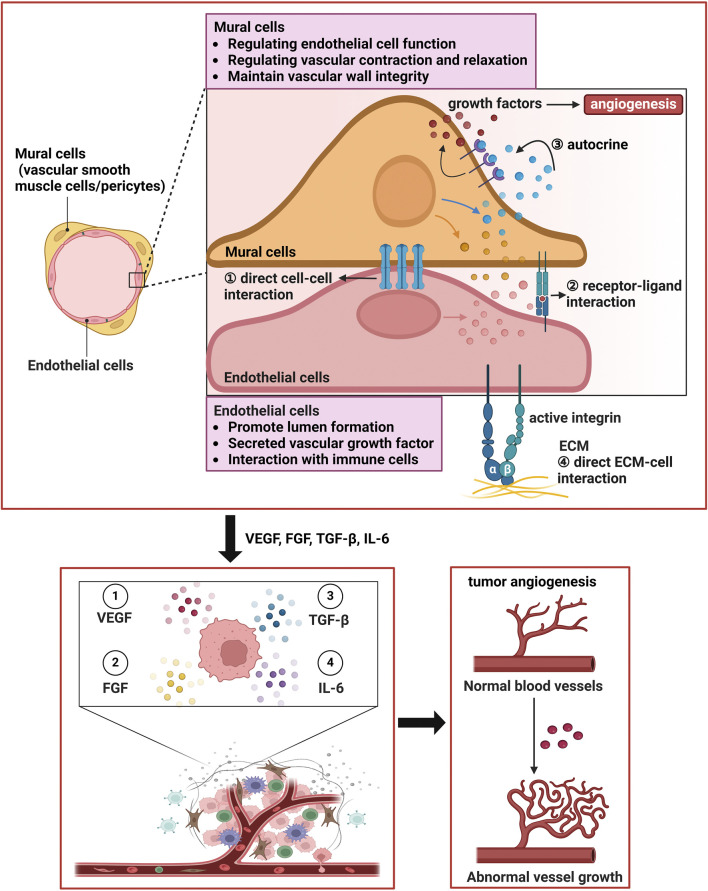
Vascular wall cells interact with endothelial cells to facilitate tumor angiogenesis. (1) Mural cells can make direct physical contact with endothelial cells to facilitate angiogenesis. (2) Vascular smooth muscle cells and pericytes can promote the production of certain growth factors through autocrine mechanisms, further enhancing angiogenesis. (3) The extracellular matrix can also promote the biological behaviors and functions of vascular smooth muscle cells and pericytes. The two types of cells work synergistically to promote tumor angiogenesis. Additionally, mural cells play important roles in regulating endothelial cell function, modulating vascular contraction and relaxation, maintaining the integrity of the vascular wall, and promoting ECM production to form the vascular system. In addition, endothelial cells play key roles in lumen formation, the secretion of vascular growth factors, interactions with immune cells, and autocrine signaling.

Additionally, angiogenesis involves interactions between cells, such as signalling between vascular smooth muscle cells (VSMCs)/pericytes and endothelial cells (ECs), which are crucial for the stability and function of new blood vessels (Naderi-Meshkin et al., 2023; Figure 1). Recent studies have reported that pericytes, in addition to maintaining vascular integrity, participating in angiogenesis, and regulating blood flow, can also serve as reservoirs for multipotent stem/progenitor cells in white, brown, and bone marrow adipose tissues (Picoli et al., 2024). Owing to the complexity of this cell population, the identification and characterization of pericytes have been challenging. A comprehensive understanding of pericyte heterogeneity may increase the potential of pericytes as therapeutic targets for metabolic syndrome or tumor-related diseases. Cadherins are adhesion molecules that mediate Ca2+-dependent homophilic interactions controlling morphogenesis and tissue remodelling. Recent studies have shown that pericytes also express T-cadherin, which is an atypical glycosylphosphatidylinositol-anchored member of the superfamily previously associated with neuronal process guidance, endothelial angiogenic behavior, and the regulation of smooth muscle cells (Yulis et al., 2018). Dasen et al. (2023) reported that T-cadherin is a novel regulator of pericyte function and supported its necessity for pericyte proliferation and invasion during the active phase of angiogenesis. The loss of T-cadherin causes pericytes to transition to a myofibroblast state, rendering them unable to regulate endothelial angiogenic behavior. Other studies have shown that mechanical factors, such as shear stress and the physical properties of the extracellular matrix, can also influence endothelial cell behavior, further promoting angiogenesis (Kretschmer et al., 2021; Kaur and Royal, 2024).

### 2.2 Glucose metabolism and the Warburg effect in HBs and their relationship to angiogenesis

Recent studies have reported that the metabolism of endothelial cells and angiogenic growth factors jointly promote angiogenesis (Eelen et al., 2018). Budding is a fundamental characteristic of angiogenesis and refers to the transition of the EC phenotype from a quiescent state to a highly active, proliferative, and migratory state under the stimulation of proangiogenic VEGF-A (Uemura et al., 2021). Therefore, a significant amount of energy is required during angiogenesis to support the proliferation and migration of ECs. Recently, the metabolism of ECs, particularly glycolytic metabolism, has been recognized as a driving force for angiogenesis (Li et al., 2019). Our team’s previous research revealed that glucose metabolism may play an important role in the pathogenesis of infantile hemangioma (IH) (Yang K. et al., 2021). 6-Phosphofructo-2-kinase/fructose-2,6-bisphosphatase 3 (PFKFB3) is a metabolic enzyme that converts fructose-6-phosphate into fructose-2,6-bisphosphate, the most effective allosteric activator of the rate-limiting enzyme phosphofructokinase-1 (PFK-1). The knockdown of PFK-1 can inhibit angiogenesis and reduce glycolytic flux in hemangioma-derived endothelial cells (HemECs). Additionally, we found that inhibiting PFKFB3 can suppress IH angiogenesis and induce cell apoptosis, suggesting that targeting PFKFB3 may be a new therapeutic strategy for IH (Yang et al., 2023). Metabolic dysfunction of ECs or excessive angiogenesis can lead to vascular diseases, such as diabetes (Eelen et al., 2018) and pulmonary hypertension (Sutendra and Michelakis, 2014). PFKFB3 and PFKFB4 belong to the 6-phosphofructo-2-kinase/fructose-2,6-bisphosphatase (PFKFB) family, which are key regulatory enzymes in glycolysis and the pentose phosphate pathway. Their functions are both similar and complementary (Kotowski et al., 2021). PFKFB3 primarily catalyzes the synthesis of fructose-2,6-bisphosphate (F2,6BP), enhancing glycolytic flux and promoting the rapid proliferation of tumor cells. PFKFB3 is expressed at low levels in normal tissues but is significantly upregulated in various cancers (such as breast cancer and colorectal cancer), which is correlated with tumor invasiveness and poor prognosis. PFKFB3 expression is also associated with epithelial-mesenchymal transition (EMT). Research by Gu et al. (2017) indicated that knockdown of PFKFB3 not only inhibited the invasiveness of CNE2 human nasopharyngeal carcinoma cells but also suppressed the upregulation of E-cadherin while downregulating the surface expression of vimentin and N-cadherin, emphasizing the important role of PFKFB3 in cancer cell proliferation and invasiveness and suggesting that it is a potential clinical biomarker for angiogenesis and a therapeutic target for inhibiting angiogenesis. PFKFB4 is induced under hypoxic conditions and regulates the metabolic flow of glycolysis and the pentose phosphate pathway by balancing the synthesis and degradation of F2,6BP, supporting redox homeostasis and the biosynthetic needs of tumor cells. PFKFB4 has been confirmed as a key driver of metastasis in hepatoblastoma (Desterke et al., 2024). Our previous research revealed that blocking glycolysis by targeting PFKFB3 can suppress the progression of infantile hemangioma, suggesting that PFKFB3 may promote the occurrence and development of IH by enhancing angiogenesis (Yang et al., 2023). Therefore, we speculate that PFKFB3 may also promote the metastasis and progression of HB for the following reasons. First, PFKFB3 enhances glycolysis, providing energy and biosynthetic precursors (such as nucleotides and lipids) for HB cells and supporting their invasiveness and metastasis. Second, PFKFB3 is highly expressed in endothelial cells and promotes tumor angiogenesis by activating the HIF-1α/VEGF pathway. Experiments have shown that inhibiting PFKFB3 can reduce endothelial cell migration and vascular network formation. Third, in HBs, PFKFB4 may regulate metabolic adaptation under hypoxic conditions, while PFKFB3 maintains basal glycolytic levels, together promoting angiogenesis and metastasis in the TME. In breast cancer studies, coexpression of PFKFB3 and PFKFB4 was significantly associated with metastasis and chemotherapy resistance, suggesting that they may drive tumor progression through shared signalling pathways (such as the PI3K/AKT pathway). In future studies, we will knock down PFKFB3 in HB cells and animal models to observe its effects on angiogenesis and metastasis and compare its effects with those of the PFKFB4 knockout phenotype. Additionally, developing dual inhibitors (small molecule inhibitors) may be more effective in blocking the metabolic adaptation and metastasis of HB.

The Warburg effect refers to the phenomenon in which tumor cells prefer to produce energy through glycolysis rather than mitochondrial oxidative phosphorylation, even when oxygen is present, accompanied by high lactate secretion. This metabolic characteristic has been shown in HBs, where HB cells express high levels of key glycolytic enzymes and glucose transporters, which leads to increased glucose uptake and lactate production (Hirschhaeuser et al., 2011). Additionally, some studies have shown that genes related to mitochondrial metabolism (such as PPARα) are downregulated in HBs, further reinforcing glycolytic dependency (Cairo et al., 2008). Most importantly, the activation of pathways such as the HIF-1α, c-Myc, and PI3K/AKT/mTOR pathways can promote the expression of glycolysis-related genes, which drives the Warburg effect (Bögel et al., 2024; Liao et al., 2024). Angiogenesis is a key step in tumor growth, and the Warburg effect helps this process in several ways: (1) Lactate generated by glycolysis is released outside the cell through monocarboxylate transporters (MCTs), making the tumor microenvironment more acidic, activating HIF-1α in endothelial cells, inducing the expression of VEGF, and promoting angiogenesis (Végran et al., 2011). (2) HIF-1α/VEGF signalling axis: Even under normoxic conditions, metabolic byproducts linked to the Warburg effect (such as succinate) can stabilize the HIF-1α protein, directly increasing VEGF transcription and enhancing angiogenic capacity (Semenza, 2013). (3) Immune evasion and vascular support: Lactate can also inhibit immune cell functions (such as T-cell activation) while stimulating tumor-associated macrophages to secrete proangiogenic factors (such as IL-8), creating an immune-suppressing environment that promotes angiogenesis (Colegio et al., 2014). Some studies have shown that specific anti-VEGF treatments inhibit neovascularization and significantly suppress tumor growth in HB, with surviving vascular systems showing dilation and increased vascular smooth muscle; anti-VEGF drugs could be promising therapeutic alternatives for children with HB (McCrudden et al., 2003). In summary, the Warburg effect in HBs significantly promotes angiogenesis through metabolic reprogramming and microenvironment acidification, activating pathways such as the HIF-1α/VEGF pathway. Targeting key glycolytic molecules alone or in combination with antiangiogenic therapies could be promising strategies. Future research should further explore the molecular mechanisms of the interaction between metabolism and the microenvironment to improve clinical treatment options.

### 2.3 Metabolism and angiogenesis in HB

Recently, Monge et al. (2024) used single-cell sequencing and KEGG enrichment analysis to find that 59 metabolic enzymes were significantly upregulated in HB tumor cells, involving amino acid metabolism, carbohydrate metabolism, carbon monoxide metabolism and steroid metabolism. Among them, DNMT3B influences epigenetic modifications (DNA methylation) by regulating methionine metabolism. The developed metabolic score distinguished tumor cells from normal liver cells at the single-cell level, with an AUC of 0.98, and the metabolic score of quiescent tumor cells in the G1 phase was greater, suggesting that metabolic activity is related to tumor stem cell characteristics. These findings indicate that the activated metabolic transcriptional program has a potential impact on the epigenetic functions observed in HBs and was confirmed at the single-cell level. Previous studies have shown that reducing glutamine production can affect cell viability in the embryonal subtype of HB, but high expression of GLUL is associated with better overall survival (Schmidt et al., 2021). Additionally, CTNNB1 mutations promote metabolic reprogramming through the Wnt/β-catenin pathway (Liu et al., 2022), and metabolic reprogramming may promote angiogenesis through the following mechanisms: (1) Lactate produced by the Warburg effect can induce HIF-1α, which upregulates proangiogenic factors such as VEGF. (2) Folate cycle products support endothelial cell proliferation. This study revealed the metabolic‒epigenetic regulatory role of DNMT3B, while DNMT inhibitors (such as 5-azacytidine) can inhibit angiogenesis in solid tumors (Camacho et al., 2023). These findings suggest that future research could explore whether DNMT3B regulates the expression of proangiogenic genes (such as VEGF) through methylation. Currently, multiple studies indicate that high expression of VEGF and angiopoietin-2 (ANGPT2) in HB is associated with increased vascular density. Future research should combine spatial metabolomics with dynamic monitoring of angiogenic factors to elucidate the specific mechanisms of the HB metabolic-angiogenesis axis. Moreover, targeting “dual-effect targets”, such as DNMT3B, which has both metabolic and epigenetic regulatory functions, may provide a theoretical basis for the development of new combined therapeutic strategies.

### 2.4 Inflammatory response and angiogenesis

Under physiological and pathological conditions, inflammatory cells produce cytokines and other stimulants that promote or inhibit neovascularization (Peluzzo and Autieri, 2022). According to genetic analysis, tumor-associated macrophages (TAMs) can produce various molecules, including VEGF, tumor necrosis factor-α (TNF-α), interleukin-1β (IL-1β), interleukin-8 (IL-8), platelet-derived growth factor (PDGF), thymidine phosphorylase, and MMPs involved in tumor angiogenesis; TAMs promote direct tumor angiogenesis by releasing proangiogenic factors (such as VEGF-A) in hypoxic regions of tumors. These factors play a role in tumor angiogenesis (Wang et al., 2021). Additionally, IL-8 signalling can promote their migration, invasion, and capillary formation through the expression of matrix metalloproteinase-2 (MMP-2) and matrix metalloproteinase-9 (MMP-9) (Flores-Pliego et al., 2023). The formation of tumor blood vessels requires macrophages expressing tyrosine kinase (TIE2) and promotes tumor angiogenesis. Tumor hypoxia upregulates the expression of the TIE2 receptor on the TME and the production of angiopoietin-2 (ANG-2) in tumor endothelial cells (TECs) (Joussen et al., 2021). ANG-2 may recruit monocytes expressing TIE2 to tumors and inflammatory sites, where ANG-2 binds to TIE2, promoting the production of angiogenic factors.

Furthermore, they initiate angiogenesis by destabilizing existing blood vessels (Saharinen et al., 2017). Therefore, inflammation during tumor angiogenesis is related primarily to the recruitment of TAMs from circulating monocytes. During the recruitment process, chemokines released by tumor cells or TECs induce chemotaxis. The recruited TAMs promote tumor angiogenesis directly by releasing proangiogenic factors or indirectly by releasing cytokines. Thus, antiangiogenic therapies targeting tumor inflammation may be powerful options for cancer treatment (Saharinen et al., 2008). In our previous review (Ji et al., 2020), we reported the upregulation of ANG-2 expression in patients with kaposiform hemangioendothelioma, suggesting that the reason may be the activation of TIE-2 induced by ANG-2, triggering Akt/mTOR signalling, which is primarily mediated by the PI3K alpha catalytic subunit (PIK3CA) in endothelial cells (Kim I. et al., 2000). Therefore, the formation of blood vessels in HBs may also be related to the ANG/TIE2 pathway, but extensive experiments are needed to verify this hypothesis.

### 2.5 Interaction between angiogenesis and the TME

**FIGURE 2:**
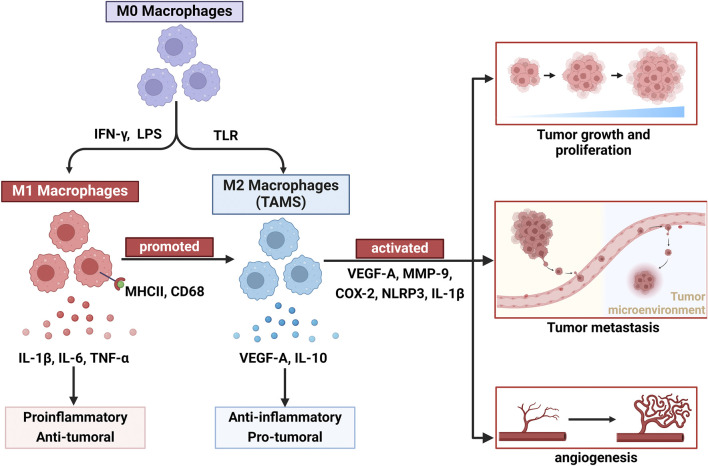
Macrophage plasticity and polarization dynamics: dual roles of M1 and M2 subtypes in tumor angiogenesis. Owing to the plasticity of macrophages, undifferentiated macrophages (M0) can differentiate into two types: classically activated macrophages (M1) and alternatively activated macrophages (M2). M1 macrophages are stimulated by IFN-γ and bacterial lipopolysaccharides, exert proinflammatory and antitumor vascular effects, and express IL-1β, IL-6, and TNF-α. Additionally, under certain conditions, M1 macrophages can also be converted to M2 macrophages to promote tumor angiogenesis. M2 macrophages, which are stimulated by certain growth factors and inflammatory factors (VEGF-A, MMP9, COX-2, NLRP3, and IL-1β), play roles in promoting tumor growth, proliferation, metastasis, and angiogenesis and producing IL-10 and TGF-β.

The TME has a profound impact on angiogenesis, with tumor cells promoting the formation of surrounding blood vessels through the secretion of various factors, such as VEGF-A. This process facilitates tumor growth and metastasis (Jiang et al., 2020). Additionally, immune cells, fibroblasts, and extracellular matrix components within the TME influence the dynamic balance of angiogenesis through complex interactions (Yang F. et al., 2024). Macrophages are important components of the mononuclear phagocyte system and are involved in immune system regulation, pathogen clearance, wound healing, and angiogenesis (Guilliams and Scott, 2022). TAMs can secrete proangiogenic factors that increase tumor vascularization, thereby supporting tumor growth and metastasis (Lazarov et al., 2024). Under stimulation by various cytokines, macrophages can polarize into two forms that exhibit different functions: M1 macrophages, which are proinflammatory and inhibit tumor growth, and M2 macrophages, which are anti-inflammatory and promote tumor growth (Gao et al., 2022; Figure 2). However, recent studies have shown that the role of M1-like TAMs in tumors is bidirectional. CD68^+^ human leukocyte antigen DR + M1-like TAMs have been shown to increase the motility of tumor cells in hepatocellular carcinoma (Wang H. et al., 2014). Furthermore, the relationship between M1-like macrophages and tumor metastasis may be partially attributed to the influence of inflammatory cytokines, such as IL-1β, TNF-α, and IL-6, which can directly or indirectly promote the proliferation of endothelial cells (Nakao et al., 2005). Therefore, understanding the interactions between angiogenesis and the TME is crucial for developing new antitumour therapeutic strategies.

Angiogenesis in the TME also significantly impacts tumor metastasis. First, newly formed blood vessels provide abundant oxygen and nutrients to tumor cells, promoting their proliferation and survival (Yuan et al., 2021). Second, angiogenesis creates “escape” routes for tumor cells during metastasis. Research indicates that under hypoxic conditions, tumor cells can promote angiogenesis and metastasis by secreting exosomes, a process regulated by miR-3174, suggesting that alterations in the microenvironment directly affect the metastatic potential of tumor cells (Cabus et al., 2021). Furthermore, other cellular components in the microenvironment, such as fibroblasts and immune cells, influence the metastatic process by secreting cytokines and modulating angiogenesis (Stewart et al., 2016). Consequently, changes in angiogenesis within the microenvironment not only affect tumor growth but also play a pivotal regulatory role in metastasis, providing new avenues for cancer treatment.

## 3 Angiogenesis in HBs

### 3.1 Significance of angiogenesis and tumor hypoxia status in HB

The importance of angiogenesis in tumor progression is mainly evident in the following ways: (1) Nutrient and oxygen supply (Terashima et al., 2013): the growth of solid tumors requires oxygen and nutrients through angiogenesis. VEGF is the key player in angiogenesis, and its expression is regulated by hypoxia, oncogene activation, and inflammatory factors (such as IL-6 and TNF-α). Even though the unusual structure of tumor blood vessels can temporarily break from hypoxia, it can lead to disrupted blood flow, further exacerbating metabolic stress (Folkman, 1971). (2) Clearance of metabolic waste: Tumour cells churn out a large amount of lactic acid via the Warburg effect, which is pushed out of the cell via monocarboxylate transporters 1/4 (MCT1/4). Newly formed blood vessels not only clear lactic acid but also promote endothelial cell migration by activating G protein-coupled receptor 1 (GPR81), forming a feedback loop that promotes angiogenesis (Carmeliet and Jain, 2011). (3) Metastatic pathways: The abnormal structure of the tumor neovasculature (such as loose connections between endothelial cells and insufficient pericyte coverage) creates physical routes for circulating tumor cells (CTCs) to invade. Additionally, the PDGF and TGF-β that leak from blood vessels can trigger EMT, enhancing the metastatic ability of tumor cells (Weis and Cheresh, 2011). In HB, this biological process is particularly critical, as this type of tumor is characterized by high vascularization and often occurs in the metabolically active liver environment of infants (Kim M. S. et al., 2000). (4) Treatment resistance: The high interstitial fluid pressure (IFP) resulting from excessive vascularization makes it difficult for chemotherapy drugs to pass through, while hypoxic regions significantly reduce radiotherapy sensitivity by activating HIF-1α-dependent multidrug resistance genes and DNA repair pathways. Anti-angiogenic drugs (such as bevacizumab) can temporarily improve drug delivery through a “vascular normalization window,” but using them for a long time might lead to a more aggressive tumor type (Jain, 2014).

The oxygen partial pressure gradient in the tumor microenvironment finely regulates angiogenesis. Under normoxic conditions, tumor cells maintain vascular homeostasis through baseline levels of VEGF, while hypoxic conditions significantly increase angiogenic capacity by activating both the hypoxia-inducible factor (HIF) pathway and the aryl hydrocarbon receptor (AhR) pathway (Jacquemin et al., 2024). In HepG2 cells, glucose deprivation has been shown to induce AhR nuclear translocation, directly upregulating VEGF expression through the activation of the transcription factor ATF4, a mechanism that allows tumors to maintain angiogenesis under metabolic stress (Bai et al., 2020). Notably, the low-oxygen environment in HBs might have several unique health implications, as studies have shown that tumor cells reconstruct metabolic networks under HIF activation: increased expression of glycolytic enzymes accompanies mitochondrial network fragmentation. This shift in metabolism, while slowing down cell growth, significantly enhances cell survival under metabolic stress. This adaptive mechanism may explain why hepatoblastoma can maintain cell viability in hypoxic regions and provide continuous stimulation for subsequent angiogenesis.

Previous studies have shown that natural compounds such as Torilin can effectively inhibit endothelial cell proliferation and lumen formation by downregulating VEGF and insulin-like growth factor-II (IGF-II) expression in hepatoblastoma cells, resulting in strong antiangiogenic effects in animal studies (Kim M. S. et al., 2000). Additionally, hypoxia can maintain HIF-1α stability by blocking prolyl hydroxylases (PHDs), driving the expression of genes such as VEGF and glucose transporter-1 (GLUT1) and promoting angiogenesis and the Warburg effect while also upregulating PD-L1 and adenosine signalling to induce immune suppression. In HB, CTNNB1 mutations synergistically increase VEGF-A/ANGPT2 expression through β-catenin signalling, leading to abnormal increases in vascular density, with its unique metabolic‒hypoxic malignant cycle manifesting as an increase in lactic acid production, activating the endothelial cell HIF-1α/VEGF pathway through MCT4 transport, forming a positive feedback loop of “glycolysis--acidification--angiogenesis”. Therefore, future combined strategies targeting the hypoxia-metabolism-angiogenesis axis could significantly inhibit the progression of HB. Furthermore, spatial multiomics technologies can help determine how metabolic enzymes (pyruvate kinase M2 (PKM2) and lactate dehydrogenase (LDHA)) are related to oxygen gradients, locating metabolic hotspots that promote angiogenesis (Monge et al., 2024).

### 3.2 Relationships between angiogenesis and HB stage and prognosis

The degree of angiogenesis is closely related to the stage of HB. Studies have shown significant differences in angiogenesis among different stages of HB (Xu et al., 2024). Early-stage HB is usually associated with lower levels of angiogenesis, while there is a significant increase in angiogenesis as the tumor progresses, especially when it reaches advanced stages. This phenomenon may be related to the ability of tumor cells to adapt to hypoxic environments; under hypoxic conditions, tumor cells secrete factors such as VEGF-A to stimulate angiogenesis, thereby improving their survival environment. High-stage HB is typically associated with increased vascular density and increased angiogenic capacity, which are closely related to tumor aggressiveness and metastatic potential. Furthermore, the level of angiogenic activity can serve as an indicator of the prognosis of HB patients. Research shows that high expression of angiogenesis-related factors in HB patients is often associated with poor prognosis (Chen et al., 2017). This may be because enhanced angiogenesis promotes the supply of nutrients and oxygen to tumor cells, supporting rapid tumor growth and metastasis. Additionally, angiogenesis may affect the TME, promoting immune evasion mechanisms and further exacerbating the malignancy of tumors. Therefore, assessing the characteristics of angiogenesis in HB not only helps in understanding the biological behavior of the tumor but also may provide an important reference for clinical staging and prognosis assessment. Further research on the relationship between angiogenesis and the HB stage may provide a theoretical basis for the development of new treatment strategies.

### 3.3 Association between angiogenesis and HB metastasis

Angiogenesis is a key process in tumor growth and metastasis, particularly in HB, where its metastatic ability is closely related to angiogenesis in the TME. Studies have shown that HB cells can promote the formation of new blood vessels by secreting various proangiogenic factors (such as VEGF-A and FGF-19), thereby providing the necessary oxygen and nutritional support for tumor growth and metastasis (Dong et al., 2017; Elzi et al., 2016). lncRNAs are involved in many biological processes, such as angiogenesis, invasion, cell proliferation, and apoptosis. They play a crucial role in the pathophysiology of HB. Dong et al. (2017) reported that CRNDE was significantly upregulated in human HB specimens and metastatic HB cell lines. In *in vivo* experiments, CRNDE knockdown inhibited tumor growth and angiogenesis and reduced HB cell viability, proliferation, and *in vitro* angiogenic activity, suggesting that CRNDE could serve as a diagnostic marker and therapeutic target for HB. Dong et al. (2016) reported that the expression level of TUG1 was significantly increased in liver samples from HB patients and metastatic HB cell lines. The overexpression of TUG1 promoted increased expression of VEGFA, enhancing the growth of blood vessels in HBs, whereas the knockdown of TUG1 significantly reduced tumor angiogenesis, thereby inhibiting tumor growth. Furthermore, they reported that the TUG1/miR-34a-5p/VEGFA signalling axis could be a candidate target for HB treatment. In addition, the lncRNA TUG1 enhances the angiogenic capacity of HBs by regulating the miR-204-5p/JAK2/STAT3 axis, which may provide favourable conditions for the metastasis of tumor cells (Yuan et al., 2021). Gong et al. (2022) reported that the protein expression of Yap was closely related to angiogenesis and prognosis in HB, potentially promoting tumor angiogenesis and metastasis through VEGF, making it a potential adverse prognostic marker.

Piezo1 is a widely expressed membrane mechanotransduction protein involved in the migration of tumor cells. In the early stages of tumors, as epithelial cell morphology changes and cellular homeostasis is disrupted, Piezo1 can promote the migration of ECs by activating calcium ion channels, thereby driving embryonic angiogenesis (Wang et al., 2019). It has been reported that the level of hypoxia-inducible factor-1α (HIF-1α) is significantly elevated in hypoxic tumor cells (Xu et al., 2016). HIF-1α, a calcium ion-sensitive factor, has also been confirmed to participate in tumor cell metastasis by promoting epithelial‒mesenchymal transition (EMT) and angiogenesis (Chen et al., 2017). Ye et al. (2022) recently reported that Piezo1 is highly expressed in HB tissues and is associated with poor prognosis in HB patients. Piezo1 overexpression promoted the expression of HIF-1α, thereby increasing VEGF expression and further promoting the metastasis of HB cells. Therefore, Piezo1 may be a potential therapeutic target for HB metastasis.

Tumor metastasis is one of the important reasons for poor prognosis in cancer patients, and angiogenesis plays a key role in this process. Tumor cells can obtain the necessary oxygen and nutrients by promoting angiogenesis, thereby supporting their growth and spread. Research indicates that tumor cells can drive angiogenesis by secreting various proangiogenic factors (VEGF-A and HIF-1α), thereby forming a specific microenvironment that promotes tumor metastasis (Chang et al., 2022). Some studies have shown that the transient potential receptor vanilloid 4 (TRPV4) channel in endothelial cells plays an important regulatory role in tumor growth and metastasis, as it regulates tumor angiogenesis and vascular integrity, inhibiting tumor growth and metastasis (Kanugula et al., 2021). Additionally, exosomes derived from cancer cells play a significant role in the metastasis process by regulating related signalling pathways and promoting angiogenesis and tumor metastasis (Olejarz et al., 2020). Zhang et al. (2021) reported that the secretion of the extracellular vesicle high-mobility group box 3 (HMGB3) by nasopharyngeal carcinoma cells could promote tumor metastasis by inducing angiogenesis, providing a new target for tumor metastasis. Furthermore, the role of platelets in the TME cannot be overlooked, as they enhance the metastatic ability of tumor cells by releasing proangiogenic factors and cytokines (Huong et al., 2019). Therefore, understanding the role of angiogenesis in the metastasis process not only helps to reveal the mechanisms of tumor metastasis but also provides potential directions for the development of new therapeutic strategies.

## 4 Regulatory factors and signalling pathways related to HB vasculogenesis

### 4.1 Wnt/β-catenin signalling pathway

The Wnt/β-catenin signalling pathway plays crucial roles in tumorigenesis, tissue homeostasis, angiogenesis, and carcinogenesis (Zhang et al., 2023). During tumor angiogenesis, overactivated Wnt/β-catenin signalling continuously induces the upregulation of proangiogenic factors (Garnier et al., 2016). Recent research by Hong et al. (2022) revealed that CD248 regulates Wnt signalling in pericytes, promoting angiogenesis and tumor growth in lung cancer. In most HBs, missense mutations or exon 3 deletions in β-catenin-encoding genes have been reported, and HBs are associated with abnormal activation of the β-catenin pathway (Bell et al., 2017). The Wnt pathway is activated by the inhibition of β-catenin degradation; thus, the level of β-catenin increases, and the protein translocates to the nucleus, where it promotes cell proliferation. Wnt3a can activate the extracellular signal regulated kinase (ERK) pathway through Ras, Raf, and mitogen-activated protein kinase (MEK), which are involved in cell proliferation and activate p38 and mitogen-activated protein kinase (MAPK), thereby promoting tumor progression (Yun et al., 2005). Some studies have shown that this pathway promotes vessel formation by regulating the proliferation and migration of endothelial cells. The activation of Wnt signalling can increase the survival of endothelial cells and promote their migration to sites of angiogenesis, thereby facilitating the formation of new blood vessels (Horitani and Shiojima, 2024). Furthermore, the Wnt/β-catenin signalling pathway interacts with other signalling pathways in the TME, collaboratively regulating angiogenesis (Kasprzak, 2020). In the TME, the upregulation of Wnt signalling is closely related to tumor progression and metastasis, further promoting tumor growth by affecting angiogenesis (Song et al., 2019). Additionally, studies have shown that inhibition of the Wnt/β-catenin signalling pathway can effectively reduce tumor cell proliferation and invasiveness, suggesting the potential application value of this pathway in tumor therapy (Yu et al., 2022).

More importantly, the Wnt/β-catenin signalling pathway plays a crucial role in the angiogenesis and progression of HB. Its abnormal activation not only promotes the proliferation and stem cell characteristics of tumor cells but also regulates angiogenesis through various mechanisms, thereby facilitating tumor development (Sha et al., 2019). Studies have shown that approximately 80% of HB patients have mutations in the CTNNB1 gene, leading to increased stability of the β-catenin protein and persistent nuclear translocation (Gupta et al., 2012). β-catenin activates the mTORC1 signalling pathway by binding to Yes-associated protein 1 (YAP1), promoting the expression of the amino acid transporter SLC38A1, which enhances tumor metabolism and growth (Molina et al., 2019). Additionally, β-catenin can directly upregulate the expression of angiogenic factors such as VEGF-A, ANGPT2, and MMP9, promoting the proliferation and migration of endothelial cells and activating the endothelial Wnt/β-catenin pathway through paracrine mechanisms, further promoting angiogenesis (Wang L. et al., 2024).

The Wnt/β-catenin pathway also enhances glycolysis by activating LDHA and PKM2, leading to lactate accumulation, which indirectly promotes VEGF expression by stabilizing HIF-1α, thus forming a positive feedback loop of “metabolism-angiogenesis” (Wang F. et al., 2024). The activation of this pathway is associated not only with the proliferation of endothelial cells in the tumor microenvironment but also with the maintenance of tumor cell survival by inhibiting TNF-α-dependent apoptosis, further promoting vascular formation. Finally, in terms of key mechanisms of tumor progression, the cooperation between β-catenin and the ERK1/2 pathway promotes EMT, inducing the expression of related factors such as SNAIL and vimentin and enhancing the invasiveness and metastatic ability of tumors (Peng et al., 2022). Targeting this signalling pathway (with inhibitors) or combining it with antiangiogenic therapy may provide new precision treatment strategies for HB patients. Furthermore, directly inhibiting the expression of β-catenin or combining it with the inhibition of synergistic pathways such as the YAP1 pathway can enhance antitumour effects. In a clinical study, the selective colocalization of β-catenin and YAP1 in the nucleus was identified in approximately 80% of hepatocellular carcinoma patients but not in patients with hepatocellular carcinoma or cholangiocarcinoma (Tao et al., 2014). Another experimental study indicated that the coexpression of mutant forms of β-catenin and YAP1 leads to the activation of YAP and Wnt signalling, which subsequently contributes to the development of hepatoblastoma, providing potential targets for future HB treatment (Min et al., 2019).

Numerous studies have confirmed that inhibiting Wnt/β-catenin protein signalling in hepatoblastoma has a good therapeutic effect, and many methods to inhibit this signalling pathway, including siRNAs, miRNAs, and drug formulations, have been reported (Sha et al., 2019). Sorafenib is a multitarget oral drug for cancer treatment that selectively targets certain protein receptors. The application of sorafenib in the treatment of hepatoblastoma can effectively inhibit cell viability, tumor progression, and angiogenesis (Eicher et al., 2012). Sorafenib combined with cisplatin can significantly reduce the viability of hepatoblastoma cells, making it a promising treatment option for high-risk or recurrent hepatoblastoma (Eicher et al., 2013). Despite several commonly used antiangiogenic drugs, such as sorafenib and cisplatin, new drugs are still under development to achieve a comprehensive, rational, and effective selection of combination chemotherapy regimens. Therefore, more clinical trials are needed to determine the optimal treatment for HB.

### 4.2 VEGF signalling pathway

The regulatory factors of angiogenesis include mainly growth factors, cytokines, and transcription factors. Among them, VEGF-A is one of the most important growth factors and primarily promotes the proliferation, migration, and lumen formation of endothelial cells by binding to its receptors and activating downstream signalling pathways (Vakhrushev et al., 2021). VEGF-A plays crucial roles not only in embryonic development but also in adult wound healing, tumor growth, and other pathological conditions. Research has shown that VEGF-A promotes the survival and migration of endothelial cells and enhances vascular permeability, thereby facilitating the formation of new blood vessels (Cui et al., 2020). Studies have shown that elevated levels of VEGF-A are associated with poor prognosis in various cancers; for example, in breast cancer and lung cancer patients, high expression of VEGF-A is closely related to tumor aggressiveness and metastasis (Banerjee et al., 2023; Liao et al., 2022). Additionally, the expression and activity of VEGF-A are regulated by various factors, including hypoxia, inflammatory factors, and cytokines, which are particularly important in the TME and can significantly increase VEGF-A expression levels, thereby promoting tumor angiogenesis (Ghafouri-Fard et al., 2020). In recent years, antibody drugs and small-molecule inhibitors that target VEGF-A signalling have been widely studied, revealing potential application value in cancer treatment (Mammadzada et al., 2020). Furthermore, FGF-23 also plays an important role in angiogenesis, especially in ischemic diseases, where FGF-23 can promote endogenous angiogenesis (Li et al., 2021; Vázquez-Sánchez et al., 2021; Ishii et al., 2021). Moreover, other factors, such as tumor necrosis factor-α (TNF-α) and transforming growth factor-β (TGF-β), also participate in regulating angiogenesis, affecting the function of endothelial cells and the stability of blood vessels (Yang Y. et al., 2021; Shintani et al., 2021; Kim et al., 2024). In addition to these growth factors, noncoding RNAs have also been found to be involved in regulating angiogenesis by modulating the expression of related genes (Adawy et al., 2023). Moreover, HIF-1α is activated in hypoxic environments, promoting the expression of VEGF, which further facilitates tumor vascularization to meet the oxygen and nutrient demands of tumor cells (Katayama et al., 2019). This process not only promotes tumor growth and metastasis but also provides new targets for tumor treatment (Yang X. et al., 2024). The interactions and regulatory mechanisms of these molecular regulatory factors provide an important basis for understanding the physiological and pathological processes of angiogenesis.

**FIGURE 3:**
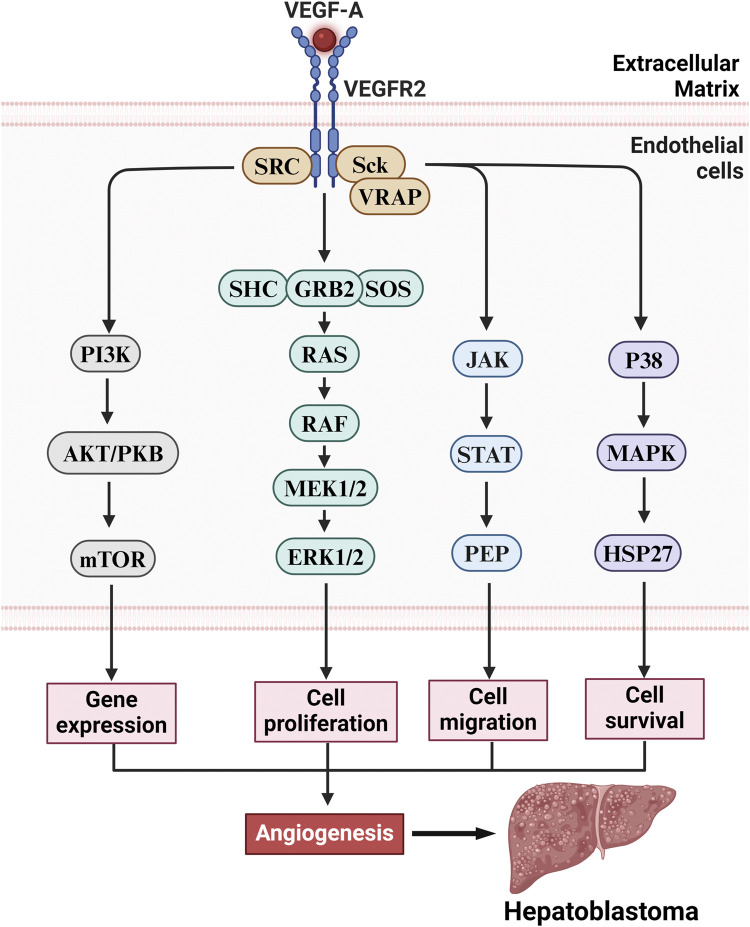
The VEGF-A/VEGFR2 axis drives HB pathogenesis via angiogenesis stimulation. Upon activation of the VEGFR2 receptor in vascular endothelial cells, VEGF-A triggers multiple downstream signaling pathways that promote angiogenesis, including gene expression, cell proliferation, migration, and survival. These pathways include the following: (1) Ras/Raf/MEK/ERK pathway: Activation of VEGFR2 initiates the Ras/Raf/MEK/ERK signaling cascade, which promotes endothelial cell proliferation and enhances angiogenesis. (2) PI3K/Akt/mTOR pathway: VEGFR2 activation leads to the activation of the PI3K/Akt/mTOR pathway, promoting cell survival and proliferation, thereby facilitating vascular formation. (3) JAK/STAT pathway: Additionally, VEGFR2 activates the JAK/STAT signaling pathway, which promotes endothelial cell proliferation and migration, further enhancing angiogenesis and facilitating tumor metastasis. (4) p38/MAPK/HSP27 pathway: VEGFR2 also activates the p38/MAPK/HSP27 signaling pathway, influencing intracellular actin reorganization and directly promoting angiogenesis. While these signaling pathways perform distinct biological functions, they exhibit synergistic effects that collectively enhance the development of tumor-associated neovascularization, ultimately driving the growth and malignant progression of HB.

Studies have shown that the characteristics of angiogenesis in HB tissue include increased microvascular density (MVD), active proliferation of endothelial cells, and upregulation of related VEGF-A (Dong et al., 2016). Some studies indicate that the main mechanisms of angiogenesis in HB include the activation of VEGF-A and its related signalling pathways (Figure 3). VEGF-A not only promotes the proliferation and migration of endothelial cells but also enhances the permeability of blood vessels, further promoting tumor growth and metastasis. Additionally, HB is also affected by epigenetic regulation, and the long noncoding RNA (lncRNA) CRNDE regulates angiogenesis in HB by targeting the miR-203/VEGF-A axis, suggesting its potential role in the progression of HB (Chen et al., 2020). Furthermore, Yuan et al. reported that the lncRNA TUG1 is upregulated in HB tissues and cells and inhibits the expression of miR-204-5p, thereby activating the Janus kinase 2 (JAK2)/signal transducer and activator of transcription 3 (STAT3) downstream signalling pathway to promote angiogenesis, further revealing the complex regulatory mechanisms of angiogenesis in HB tissue (Yuan et al., 2021). These characteristics not only provide important clues for understanding the biological behavior of HB but also offer potential targets for clinical treatment.

The VEGF signalling pathway plays a role second only to the Wnt/β-catenin signalling pathway in angiogenesis in tumors such as HB through a multistep cascade. The main mechanism involves the activation of HIF-1α or oncogenes (such as RAS) by hypoxia or inflammatory factors (such as IL-6 and TNF-α) in the tumor microenvironment, leading to the secretion of VEGF, mainly VEGF-A (Song et al., 2023). After VEGF binds to the VEGFR2 receptor on endothelial cells, it triggers receptor dimerization and tyrosine kinase autophosphorylation, activating downstream pathways such as the PI3K-AKT-mTOR pathway (which promotes cell survival and metabolism), the RAS-MAPK/ERK pathway (which drives proliferation and differentiation), and the PLCγ-PKC pathway (which regulates migration and vascular permeability). This induces endothelial cells to express integrins (such as αvβ3) and matrix metalloproteinases (MMPs) to degrade the basement membrane (Rubio et al., 2023), creating a chemotactic gradient that directs endothelial cell migration, proliferation, and differentiation into tip and stalk cells, forming a lumen structure via VE-cadherin connections, and recruiting pericytes and smooth muscle cells to finalize vascular maturation.

The overexpression of VEGF in HBs leads to abnormal vascular structures (tortuous and leaky) and creates a proangiogenic environment by mobilizing bone marrow endothelial progenitor cells (EPCs) and TAMs that secrete VEGF (Grzywa et al., 2021). This pathway is coregulated by Notch signalling and works with factors such as FGF-8 and PDGF (Wei et al., 2019), while negative regulators such as TSP-1 and interferons can reduce its activity. Clinically, anti-VEGF drugs (such as bevacizumab) inhibit angiogenesis by blocking VEGF-A, but tumors can develop resistance by upregulating FGF-2/ANG-2 or enhancing pericyte protection (Gao and Yang, 2020; Sanchez et al., 2025). In summary, VEGF activates multiple signalling pathways in endothelial cells, coordinating proliferation, migration, survival, and lumen formation, ultimately promoting physiological or pathological angiogenesis. In tumors such as HB, this process is hijacked to support tumor growth and spread, making it a promising target for new antiangiogenic therapies.

Current studies have confirmed that microRNAs (miRNAs) can inhibit tumor progression by regulating the expression of VEGF-A. miRNA-205-5p inhibits the development of renal cancer cells by negatively regulating VEGF-A expression (Huang et al., 2019). Additionally, downregulated miR-126-3p has been shown to be associated with resistance to dabrafenib in melanoma through the upregulation of VEGF-A expression (Caporali et al., 2019). Chen et al. (2017) reported that the lncRNA CRNDE promotes angiogenesis in HBs by targeting the miR-203/VEGFA axis, suggesting that this pathway plays an important regulatory role in the occurrence and development of HBs, laying the foundation for new tumor biomarkers and therapeutic targets for HB diagnosis and treatment. In-depth research on the VEGF signalling pathway can provide new targets and strategies for targeted therapy of HB.

### 4.3 PI3K/Akt signalling pathway

The phosphatidylinositol 3-kinase (PI3K)/protein kinase B (Akt) signalling pathway is widely recognized as a central regulatory factor involved in cell growth, proliferation, metabolism, angiogenesis, and metastasis (Ersahin et al., 2015). The PI3K/Akt signalling pathway plays a critical role in the occurrence and development of HB. Studies have shown that activation of the PI3K/Akt pathway is significantly associated with the proliferation, migration, and invasion capabilities of HB cells, which are activated by receptor tyrosine kinases that can be stimulated by cytokines or epidermal growth factors. The activation of this pathway promotes the activation of many intracellular proteins through phosphorylation (Garnier et al., 2016). In animal studies, the inhibition of mechanistic target of rapamycin (mTOR) affects the development and growth of HBs induced by β-catenin/Yes-associated protein 1 (YAP1) (Molina et al., 2019). Research has demonstrated that inhibiting PI3K reduces HB cell growth, accompanied by a decrease in glycogen synthase kinase-3β (GSK-3β) and Akt phosphorylation. Inhibition of PI3K increases HB cell apoptosis and decreases HB cell proliferation, which is related to an increase in p27 and a decrease in cyclin D1 levels (Hartmann et al., 2009). Recent studies by Wang et al. revealed that kin of IRRE-Like protein 1 (KIRREL) promotes gastric cancer cell proliferation and angiogenesis through the PI3K/AKT pathway, and these findings may aid in the further development of potential antiangiogenic targets (Wang T. et al., 2024).

Our team discovered in the latest research that the SOX4 protein can bind to the downstream target gene ESM1, further activating the PI3K/AKT signalling pathway, promoting angiogenesis in hemangioma endothelial cells, and ultimately facilitating the occurrence and progression of IH (Li et al., 2024). Cui et al. (2019) reported that miR-193a-5p can regulate the expression of dipeptidase 1 (DPEP1) through PI3K/Akt signalling, thereby modulating the tumorigenicity and progression of HB. The miR-193a-5p/DPEP1 signalling axis could serve as an effective therapeutic and prognostic biomarker for HB patients. Research has shown that ubiquitin-specific protease 7 (USP7) promotes the progression of HB by activating the PI3K/Akt signalling pathway, providing new insights for targeted therapy of HB (Ye et al., 2021). Additionally, activation of the PI3K/Akt pathway is associated with the chemoresistance of HB cells, and inhibiting this pathway may enhance the efficacy of chemotherapeutic drugs, thereby improving patient prognosis (Zhang et al., 2022). Research has also shown that certain natural compounds can inhibit the proliferation and metastasis of HB cells by modulating the PI3K/Akt pathway, suggesting new strategies for the treatment of HB (Zhao and Hu, 2019).

### 4.4 JAK2/STAT5 signalling pathway

The JAK2/STAT5 signalling pathway is an important intracellular signalling pathway that plays a crucial role in various biological processes, such as cell proliferation, differentiation, survival, and angiogenesis (Philips et al., 2022). Increasing evidence suggests that dysregulation of the JAK/STAT pathway is related to tumor cell proliferation, migration, invasion, and angiogenesis (Wu et al., 2023). In the liver, the suppressor of cytokine signalling 2 (SOCS2)/JAK2/STAT5 axis and the negative regulatory effect of SOCS2 are essential for maintaining the normal physiological state of hepatocytes (Masuzaki et al., 2016). Previous studies have shown that high glucose-induced JAK/STAT activation upregulates the expression of miR-181b in glomerular mesangial cells (Yang Y. et al., 2021). Our team recently reported for the first time that miR-181b-5p directly targets SOCS2 in HB. Furthermore, we found that the biological effect of miR-181b-5p on HB metastasis is mediated by the inhibition of SOCS2 and the activation of the JAK2/STAT5 signalling pathway, suggesting that miR-181b-5p may serve as a therapeutic target for HB patients (Lv et al., 2023a; Lv et al., 2023b). Additionally, recent studies have shown that inhibiting the JAK2/STAT3 signalling pathway may effectively reduce angiogenesis in HB, thereby inhibiting its metastasis (Yuan et al., 2021). In summary, a deeper understanding of the role of angiogenesis and the JAK2/STAT5 signalling pathway in the metastasis of HB is highly important for the development of new therapeutic strategies.

### 4.5 SP/NK-1R signalling pathway

Undecapeptide substance P (SP) binds to the neurokinin-1 receptor (NK-1R) to regulate cancer cell proliferation, exerts antiapoptotic effects, induces cell migration for invasion/metastasis, and promotes the proliferation of endothelial cells to generate new blood vessels (Muñoz et al., 2019). SP may promote the formation of activated epidermal growth factor receptor (EGFR) complexes after binding to NK-1R, inducing transactivation of EGFR, activating the MAPK pathway, and inducing the activation of ERK2 and deoxyribonucleic acid (DNA) synthesis, thereby promoting the progression of HB (Castagliuolo et al., 2000). Other studies have confirmed that the SP/NK-1R system can regulate the transmission of various cell signals, such as antiapoptotic signalling pathways (PI3K/Akt/mTOR), cell proliferation signalling pathways (MAPK, ERK), cell migration signalling pathways (Rho-ROCK-pMLC), the classical Wnt signalling pathway (β-catenin, c-myc, and cyclin D1), and the cyclic adenosine monophosphate (cAMP)-protein kinase A (PKA) phosphorylation signalling pathway, all of which jointly promote the progression of HB (Lim et al., 2017; Mei et al., 2013). They also reported that NK-1R antagonists reduce tumor volume and angiogenic activity in HBs. In human pancreatic xenograft models, peptide NK-1R antagonists have been shown to block tumor growth through antiproliferative and antiangiogenic mechanisms (Guha et al., 2005). This phenomenon has also been observed in HB xenograft nude mouse models (Berger et al., 2014). Therefore, the SP/NK-1R system is an important target for HB treatment, and NK-1R antagonists can serve as specific drugs that target HB cells.

## 5 Latest research progress

Antiangiogenesis therapy, as an important strategy for tumor treatment, has received widespread attention in recent years. The development of novel antiangiogenic drugs has focused mainly on targeting tumor-associated angiogenic factors and their signalling pathways. Research has shown that interventions targeting endothelial cell metabolism can significantly increase the effectiveness of antiangiogenic therapy and reduce tumor resistance (Mohammadi et al., 2022). For example, in breast cancer studies, trastuzumab deruxtecan has shown significant antitumour effects, providing new treatment options for patients (Andrikopoulou et al., 2021). Additionally, the application of nanomaterials in antiangiogenesis has also shown promising prospects; studies indicate that functionalized nanomaterials can effectively target tumor blood vessels and enhance drug delivery efficiency, thereby improving treatment outcomes (Tang et al., 2023). Furthermore, clinical trials targeting cardiovascular diseases have also demonstrated the potential application value of antiangiogenic strategies, especially in research related to improving cardiac function, suggesting that antiangiogenic therapy for the treatment of cardiovascular diseases may emerge in the future (Li and Li, 2023). These clinical studies not only validate the results of basic research but also provide more solid theoretical support for clinical practice.

The development of angiogenesis inhibitors is an important direction in current cancer treatment research. Currently, the classic targeted signalling pathways for hepatoblastoma include the PI3K/Akt and Wnt/β-catenin pathways. Compared with nonspecific targeted chemotherapy drugs, targeted drugs have high specificity and are ideal and effective treatment methods. Some targeted factors, such as PI3K, mTOR, and Wnt, are also being explored; these targeted factors can selectively interfere with tumor growth, development, and angiogenesis (Zhu et al., 2022). The PI3K/Akt pathway is involved in the occurrence of hepatoblastoma. When proteins involved in this signalling pathway are directly targeted and inhibited, the progression of hepatoblastoma can be effectively controlled (Wang S. et al., 2014). Classic targeted drugs include the PI3K inhibitor LY294002 and the mTOR inhibitor rapamycin. Some researchers have used LY294002 to treat hepatoblastoma cell lines and reported a positive reversal effect by increasing cell apoptosis and reducing cell proliferation, thereby inhibiting the growth of hepatoblastoma cells (Hartmann et al., 2009). Polyphyllin VII has strong anticancer activity against various cancers and induces autophagy and apoptosis in HepG2 cells by inhibiting the phosphorylation of PI3K, AKT, and mTOR (Zhang et al., 2016). However, the use of angiogenesis inhibitors is also associated with several side effects, such as the induction of hypertension and other complications, which must be taken seriously in clinical applications (Tsujinaka et al., 2023). Future research should focus on optimizing the use of these drugs, reducing side effects, and exploring the possibility of combination therapies to increase treatment efficacy.

Currently, many approved anti-VEGF/VEGFR or antiangiogenic drugs are in clinical use. The most widely used antiangiogenic agents include monoclonal antibodies and tyrosine kinase inhibitors (TKIs) that target the vascular endothelial growth factor (VEGF) pathway (Liu et al., 2023). For example, sorafenib has been used to treat renal cell carcinoma, hepatocellular carcinoma, and thyroid cancer; bevacizumab has been used to treat colorectal cancer, non-small cell lung cancer, and renal cell carcinoma; ranibizumab has been used to treat diabetic macular edema, age-related macular degeneration, and diabetic retinopathy; ramucirumab has been used to treat non-small cell lung cancer and gastric cancer; and sunitinib has been used to treat renal cell carcinoma and gastrointestinal stromal tumors. However, there are currently no approved anti-VEGF/VEGFR or antiangiogenic drugs for the treatment of HB. Nevertheless, some drugs approved for the treatment of other solid tumors have been explored in HB-related cell and animal experiments, with sorafenib being the most studied. It is a multitarget tyrosine kinase inhibitor (inhibiting VEGFR2/3, PDGFR, etc.) and is currently used to treat adult and pediatric hepatocellular carcinoma, but the efficacy of sorafenib as a monotherapy in HB has not been confirmed. Yang et al. (2022) found that in a hepatoblastoma nude mouse model, the combination of sorafenib with anlotinib and oxaliplatin (L-OHP) significantly inhibited tumor growth, providing a preliminary research basis for the clinical application of these drugs. Pang et al. (2021) reported that in HB cell lines, the combination of sorafenib with enhancer-binding protein (C/EBP) could inhibit tumor cell growth and migration, suggesting a promising treatment method for HB. Additionally, another drug, bevacizumab, which is a monoclonal antibody that targets VEGF-A to inhibit angiogenesis (Mebed et al., 2022), is currently in the early stages of experimental research and may become a treatment for HB in the future.

## 6 Future perspectives and conclusion

Angiogenesis plays a critical role in HB progression and is driven by complex interactions between metabolic reprogramming, oncogenic signalling (such as Wnt/β-catenin and VEGF), and TME dynamics. The molecular crosstalk between glycolysis (PFKFB3/4), the Warburg effect, and angiogenesis warrants further investigation, particularly how lactate-mediated HIF-1α stabilization and DNMT3B-driven epigenetic changes regulate proangiogenic factors such as VEGF and ANGPT2. Advanced techniques such as single-cell RNA sequencing and spatial metabolomics could reveal the metabolic heterogeneity within the TME and its spatial correlation with vascular hotspots. To develop more effective therapies, dual-target strategies that inhibit both metabolic enzymes and angiogenic pathways should be explored, with preclinical models testing combinatorial therapies such as PFKFB3 inhibitors combined with anti-VEGF agents to overcome resistance and normalize the tumor vasculature. Furthermore, the role of TAMs, particularly M2 polarization, in promoting angiogenesis via IL-8, MMPs, and ANG-2/TIE2 signalling should be further studied, with the potential for combining immunotherapies (such as TAM reprogramming or checkpoint inhibitors) with antiangiogenic drugs to disrupt immune evasion and vascular support. Genomics and proteomics could be harnessed to identify patient-specific angiogenic biomarkers for risk stratification and personalized therapy, with liquid biopsy platforms offering dynamic biomarkers such as circulating EPCs or exosomal miRNAs to monitor treatment responses. Advanced drug delivery systems, such as nanoparticles or antibody‒drug conjugates, should be engineered to improve the bioavailability of antiangiogenic agents such as sorafenib and bevacizumab, minimizing systemic toxicity while enhancing tumor-specific drug accumulation.

However, despite the existing research providing a certain knowledge base for this field, there are often differences in viewpoints and findings among different studies. For example, some studies emphasize the role of specific angiogenic factors, whereas others may focus on different signalling pathways or cell types. This difference suggests that the angiogenesis mechanism in HBs is a complex process that may be influenced by various factors. Therefore, future research should aim to integrate different research results to establish a more comprehensive model, which will help us better understand the specific mechanisms of angiogenesis in the development of HB. Furthermore, research on clinical applications remains insufficient, and we need to translate findings from basic research into effective treatment strategies. This includes the development of novel targeted therapies against angiogenesis, combined with existing treatment methods, to improve the treatment effects and prognosis of HB. Through multidisciplinary collaboration, integrating clinical, basic research, and translational medicine efforts, we hope to achieve more effective diagnosis and treatment of HB in the future.

In conclusion, the mechanism driving HB angiogenesis is very complex and includes the Wnt/β-catenin pathway, the VEGF signalling pathway and the HIF-1α-mediated hypoxia response, which promote angiogenesis and immunosuppression, complicating treatment outcomes. Anti-angiogenic therapies, while promising in preclinical settings, face challenges such as adaptive resistance and off-target effects. Integrating multiomics techniques, spatial biology, and patient-derived models will be key to uncovering HB vascular heterogeneity and identifying vulnerability to context-dependent therapy. A shift toward system-level interventions, such as targeting the metabolism‒angiogenesis axis and addressing the dual role of TAMs and pericytes in vascular remodelling, could accelerate personalized therapy. We should prioritize combination protocols in early-stage clinical trials with biomarker-oriented patient selection aimed at translating these insights into lasting treatments for patients with HB. Interdisciplinary collaboration is essential to translate these findings into effective, long-lasting treatments.

## Glossary

HB: hepatoblastoma
TME: tumor microenvironment
FGF: fibroblast growth factor
VSMCs: vascular smooth muscle cells
IH: infantile hemangioma
PFKFB3: 6-phosphofructo-2-kinase/fructose-2,6-bisphosphatase 3
PFK-1: phosphofructokinase-1
TAMs: tumor-associated macrophages
IL-1β: interleukin-1β
IL-8: interleukin-8
PDGF: platelet-derived growth factor
TIE2: tyrosine kinase
ANG-2: angiopoietin-2
TECs: tumor endothelial cells
TNF-α: tumor necrosis factor-α
HIF-1α: hypoxia-inducible factor-1α
LDHA: lactate dehydrogenase
PKM2: pyruvate kinase M2
HIF: hypoxia-inducible factor
MCT1/4: monocarboxylate transporters 1/4
lncRNA: long noncoding RNA
CRNDE: colorectal neoplasia differentially expressed
JAK2: Janus kinase 2
STAT3: signal transducer and activator of transcription 3
ECs: endothelial cells
HIF-1α: hypoxia-inducible factor-1α
VEGF-A: vascular endothelial growth factor A
miRNAs: microRNAs
SOCS2: suppressor of cytokine signalling 2
PI3K: phosphatidylinositol 3-kinase
Akt: protein kinase B
mTOR: mechanistic target of rapamycin
DPEP1: dipeptidase 1
ERK: extracellular signal regulated kinase
MAPK: mitogen activated protein kinase
YAP1: Yes-associated protein 1
NK-1R: neurokinin-1 receptor
EGFR: epidermal growth factor receptor
